# The pathophysiology of malarial anaemia: where have all the red cells gone?

**DOI:** 10.1186/1741-7015-6-24

**Published:** 2008-08-21

**Authors:** Oscar K Kai, David J Roberts

**Affiliations:** 1 Wellcome Trust/Kenya Medical Research Institute-Centre for Geographic Medicine Research Coast, Immunology Department, PO Box 230, Kilifi 80108, Kenya; 2 National Blood Service – Oxford Centre, John Radcliffe Hospital, Headington, Oxford OX3 9BQ, UK

## Abstract

Malarial anaemia is an enormous public health problem in endemic areas and occurs predominantly in children in the first 3 years of life. Anaemia is due to both a great increase in clearance of uninfected cells and a failure of an adequate bone marrow response. Odhiambo, Stoute and colleagues show how the age distribution of malarial anaemia and the haemolysis of red blood cells may be linked by an age-dependent increase in the capacity of red blood cells to inactivate complement components absorbed or deposited directly on to the surface of the red blood cell. In this commentary, we discuss what has been established about the role of complement deposition on the surface of red blood cells in the pathology of malarial anaemia, how genetic polymorphisms of the complement control proteins influence the outcome of malaria infection and how the findings of Odhiambo, Stoute and colleagues and others shed light on the puzzling age distribution of different syndromes of severe malaria.

## Commentary

In the accompanying article, Odhiambo, Stoute and colleagues show how the age distribution of malarial anaemia and the haemolysis of red blood cells (RBCs) may be linked by an age-dependent increase in the capacity of RBCs to inactivate complement components absorbed or deposited directly on to the surface of the RBC [1]. The work raises not only a number of important new lines of research but also challenges malaria researchers to apply this basic work to develop new ways to prevent and treat malaria.

Malaria remains an enormous problem in public health around the world [2]. Over 2 billion people live in malaria-endemic areas and up to 1 million children die each year of malaria. Severe falciparum malaria may present a variety of syndromes, but presents most frequently in childhood with severe malarial anaemia or coma. The difference in age distributions of children presenting with these syndromes is as striking as it is puzzling; the median age of children presenting with severe malarial anaemia is typically 1 to 3 years old, while the median age of children presenting with coma is significantly and consistently older, typically 3 to 5 years old [3].

Furthermore, there remain major unsolved problems about the fundamental pathophysiology of all syndromes of severe malaria. The rapid drop in haemoglobin during acute infection and the slower decline in chronic infection appear to be due to increased extravascular haemolysis of RBCs with a concomitant failure of the bone marrow to increase red cell production to compensate for these losses [4].

The increased clearance of infected cells is readily explained by the rupture of cells after completion of the parasite's intra-erythrocytic life cycle and opsonisation and clearance of intact infected RBCs. Rather less obvious is why and how uninfected cells are also cleared. It has been estimated that approximately 10 uninfected cells are cleared from the circulation for every infected cell and so the clearance of uninfected cells is of crucial importance for the development of malarial anaemia [5].

Why are uninfected RBCs cleared in such large numbers? Certainly the number and activation of splenic and other macrophages for phagocytosis of red cells is greatly increased during malarial infection [6-9]. The increased clearance of uninfected erythrocytes is also due to extrinsic and intrinsic changes to the RBCs that enhance their recognition and phagocytosis.

Uninfected RBCs have a reduced deformability leading to enhanced clearance in the spleen and a severe reduction in red cell deformability is also a strong predictor for mortality measured on admission, both in adults and children with severe malaria [10,11]. Second, the deposition of immunoglobulin and complement on uninfected RBCs may enhance receptor-mediated uptake by macrophages.

The role of immunoglobulin and complement in marking uninfected RBCs for clearance by phagocytes was first studied by Facer and colleagues [12,13] in The Gambia in the 1970s. It soon became clear that the Direct Coombs' Test (DCT) for immunoglobulins and/or complement deposited on the surface of RBCs was frequently positive in children with malaria [12]. The antibodies giving rise to the positive DCT were not autoimmune but were directed against malarial antigens [13] (and our unpublished observations) and may include complexes of immunoglobulin G (IgG) with malarial antigens including ring stage protein 2 [14].

The story of how absorbed immune complexes may contribute to increased clearance of uninfected RBCs lay dormant for 20 years when Waitumbi, Stoute and colleagues based in Western Kenya began to study how immune complexes caused haemolysis [15]. Appreciation of this work requires some knowledge of the control of complement deposition on the surface of RBCs.

Here, a number of proteins are involved in the control of complement activation. Complement receptor 1 (CR1 or CD35), decay accelerating factor (DAF or CD55) and the membrane inhibitor of reactive lysis (MIRL or CD59) may enhance binding of C3b in immune complexes (CR1), enhance inactivation of C3 convertases (CR1 and CD55) and interfere with the assembly of the terminal components of complement that form the membrane attack complex (CD59) [16]. Immune complexes and CR1 may then be removed by splenic macrophages and the RBCs depleted of immune complexes CR1 (CD35) and CD55 and returned to the circulation.

In previous papers, Waitumbi, Stoute and colleagues have shown that the amount of red cell surface IgG is increased but red cell surface CR1 and CD55 reduced in children with severe malaria compared with asymptomatic and symptomatic controls [15]. The difference in surface IgG levels appeared to be functionally significant as RBCs from children with severe anaemia were more susceptible to phagocytosis *in vitro *than RBCs from controls. Decline in CD35 or CR1 expression and increases in immune complexes bound on uninfected RBCs were associated with anaemia but these declines in CD35 (CR1) and CD55 expression were only transiently associated with malaria infection and levels returned to normal after infection had been cleared [17]. These data are all consistent with the ability of CD35 and CD55 to inactivate the formation of C3a and reduction in CD35 and CD55 expression when bound immune complexes are cleared by phagocytes.

How does this relate to the age-dependent incidence of malarial anaemia? Population studies in Europe and Africa showed the CR1 expression was strongly age-dependent and increases of both CR1 and CD55 were seen after 4 years of age and low levels of CR1 and CD55 expression were seen in a cases of severe malarial anaemia compared with slightly older children with cerebral malaria [18]. Odhimbo, Stoute and their colleagues now show that haemoglobin is inversely associated with increased age and percentage C3b-positive uninfected RBCs are inversely and directly associated with CR1 levels measured shortly after infection [1]. Therefore, reduction in these proteins (CR1, CD55 and CD59) may increase the susceptibility of children to malarial anaemia.

How do these findings help explain the age distribution of syndromes of severe malaria? If clearance of the immune complexes absorbed on to the surface of uninfected cells is reduced in younger children expressing lower levels of CD35 and CR1, then clearance of these cells would be increased, leading to more anaemia in these younger age groups.

Genetic polymorphisms also affect the expression levels, sequence and domain  structure of CR1 in Africans and other populations [19,20]. Moreover, CR1 is a ligand for the variant antigens expressed at the surface of infected RBCs allowing the formation of rosettes of infected and uninfected RBCs [21,22]. In Melanesian populations, low levels of CR1 expression have been associated with reduced rosette formation and protection from severe malaria [20].

It is possible therefore, that age-related and genetically determined reduction of levels of CR1 expressed on RBCs are associated with an increased susceptibility to anaemia but protection from other forms of severe malaria and may provide an example of how innate resistance to one syndrome of malaria may be at the expense of susceptibility to other pathophysiological pathways involved in malaria infection.

These hypotheses should ideally be tested in longitudinal studies using genetically and phenotypically well-characterised children to ascertain the levels of these age-dependent, complement regulatory proteins prior to infection and determine their association with haemoglobin levels during acute infection and with the incidence of severe disease through childhood. Such studies can only be done in a few sites in Africa where large populations can be followed before and after infection and presentation to a health facility using a demographic surveillance system.

In assessing the role of these age-related changes to the complement regulatory proteins in the differential presentation of anaemia and coma, one would also have to consider the recently described findings of increased levels of erythropoietin (EPO) seen in younger children with anaemia [23] and malaria [24] and the increased protective effect of EPO on survival after coma, particularly in a younger age group [25]. These alternative explanations for susceptibility to anaemia in younger age groups on one hand and increased protection from cerebral malaria or coma on the other are not mutually exclusive and it is possible, indeed likely, that many factors underpin such a well-defined difference in the clinical epidemiology of these malarial syndromes.

Finally, we would ask where these studies will lead in the quest for new methods to prevent or treat malarial infection. The findings of Odhiambo, Stoute and colleagues presented in the accompanying article in *BMC Medicine *provide us with a clearer understanding of the causes of anaemia in children with malaria. Translating this new understanding of pathology into fruitful avenues to investigate prevention and treatment of malaria is perhaps the greatest challenge of clinico-pathological studies in this and other disciplines.

## List of abbreviations

CR1: complement receptor 1; DAF: decay accelerating factor; DCT: Direct Coombs' Test; EPO: erythropoietin; MIRL: membrane inhibitor of reactive lysis; RBC: red blood cell.

## Pre-publication history

The pre-publication history for this paper can be accessed here:


